# 5-Methyl-1-(3-nitro­benz­yl)-1*H*-1,2,3-triazole-4-carboxylic acid monohydrate

**DOI:** 10.1107/S1600536808030584

**Published:** 2008-10-09

**Authors:** Jie Xiao, Wen Xiang Wang, Hong Zhao

**Affiliations:** a Ordered Matter Science Research Center, College of Chemistry and Chemical Engineering, Southeast University, Nanjing 210096, People’s Republic of China

## Abstract

The title compound, C_11_H_10_N_4_O_4_·H_2_O, was synthesized from 1-azido­methyl-3-nitro­benzene and ethyl acetyl­acetate. Single-crystal X-ray analysis reveals that the dihedral angle between the triazole and benzene ring planes is 84.80 (2)°. The packing of the mol­ecules is stabilized by strong O—H⋯O hydrogen bonds involving the solvent water mol­ecules as donors and acceptors. The resulting layers are arranged into a three-dimensional framework *via* weak C—H⋯O inter­actions.

## Related literature

For the synthesis of the title compound, see: El Khadem *et al.* (1968 ▶). For related literature about biological activity of triazole-based compounds, see: Olesen *et al.* (2003 ▶); Tian *et al.* (2005 ▶).
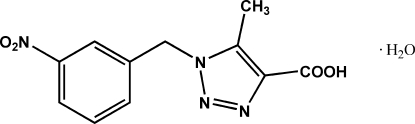


## Experimental

### 

#### Crystal data

C_11_H_10_N_4_O_4_·H_2_O
*M* *_r_* = 280.25
Triclinic, 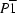

*a* = 7.1649 (14) Å
*b* = 8.2064 (16) Å
*c* = 11.281 (2) Å
α = 84.88 (3)°
β = 77.70 (2)°
γ = 89.38 (3)°
*V* = 645.5 (2) Å^3^
*Z* = 2
Mo *K*α radiation
μ = 0.12 mm^−1^
*T* = 293.1 K
0.20 × 0.18 × 0.15 mm

#### Data collection

Rigaku SCXmini diffractometer
Absorption correction: multi-scan (*CrystalClear*; Rigaku, 2005 ▶) *T*_min_ = 0.965, *T*_max_ = 0.977
6749 measured reflections
2959 independent reflections
1824 reflections with *I* > 2σ(*I*)
*R*_int_ = 0.040

#### Refinement

*R*[*F* ^2^ > 2σ(*F* ^2^)] = 0.064
*wR*(*F* ^2^) = 0.181
*S* = 1.02
2959 reflections
186 parameters
3 restraints
H atoms treated by a mixture of independent and constrained refinement
Δρ_max_ = 0.27 e Å^−3^
Δρ_min_ = −0.21 e Å^−3^

### 

Data collection: *CrystalClear* (Rigaku, 2005 ▶); cell refinement: *CrystalClear*; data reduction: *CrystalClear*; program(s) used to solve structure: *SHELXS97* (Sheldrick, 2008 ▶); program(s) used to refine structure: *SHELXL97* (Sheldrick, 2008 ▶); molecular graphics: *SHELXTL* (Sheldrick, 2008 ▶); software used to prepare material for publication: *SHELXTL*.

## Supplementary Material

Crystal structure: contains datablocks I, global. DOI: 10.1107/S1600536808030584/bh2192sup1.cif

Structure factors: contains datablocks I. DOI: 10.1107/S1600536808030584/bh2192Isup2.hkl

Additional supplementary materials:  crystallographic information; 3D view; checkCIF report

## Figures and Tables

**Table 1:** Hydrogen-bond geometry (Å, °)

*D*—H⋯*A*	*D*—H	H⋯*A*	*D*⋯*A*	*D*—H⋯*A*
C4—H4*A*⋯O4^i^	0.96	2.52	3.267 (3)	135
O1*W*—H1*A*⋯O1^ii^	0.85	1.94	2.755 (3)	161
O1*W*—H1*B*⋯N1^iii^	0.93	1.95	2.846 (3)	160
C11—H11⋯O3^iv^	0.93	2.57	3.423 (4)	153
O2—H2⋯O1*W*^iv^	0.77 (5)	1.88 (5)	2.592 (3)	153 (6)

Symmetry codes: (i) 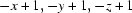; (ii) 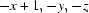; (iii) 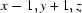; (iv) 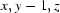.

**Fig. 1.**
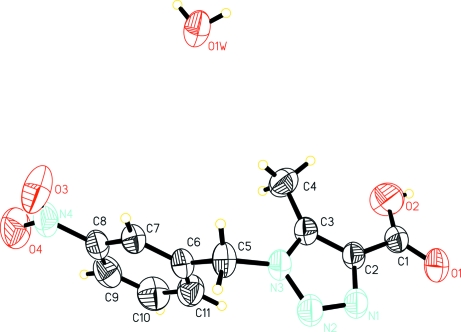
The molecular structure of (I), showing the atomic numbering scheme. Displacement ellipsoids are drawn at the 50% probability level.

**Fig. 2.**
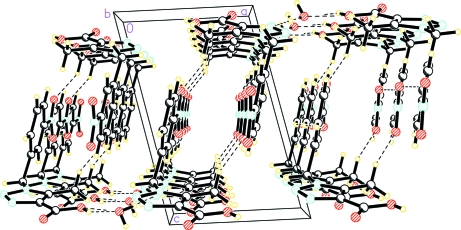
Packing diagram of the title compound, showing the crystal structure along the b axis.

## supplementary crystallographic information

### Comment

It is well known that many triazole-related molecules have received much attention due to their biological activities (Olesen *et al.*, 2003; Tian *et al.*, 2005). We report herein the crystal structure of the title compound, (I, Fig. 1). The bond lengths and angles have normal values. The dihedral angle between the triazole and phenyl planes is 84.80 (2)°. The packing of the molecules is stabilized by strong hydrogen bonds (Table 1) between the triazole molecules and lattice water molecules. Meanwhile, the 0D discrete molecules are arranged into a three-dimensional framework *via* hydrogen bond interactions and weak contacts (Fig. 2).

### Experimental

The title compound was prepared from 1-(azidomethyl)-3-nitrobenzene, according to the reported method (EI Khadem *et al.*, 1968), in 70% yield. Colourless prisms of (I) were obtained by slow evaporation of a 95% ethanol/water solution at room temperature.

### Refinement

All H atoms were detected in a difference map. Nevertheless, all C-bonded H atoms were placed in calculated positions, and constrained in a riding motion approximation with fixed bond lengths and *U*_iso_ parameters: C_aryl_—H = 0.93 Å, with *U*_iso_(H) = 1.2*U*_eq_(C); C_methyl_—H = 0.96 Å, with *U*_iso_(H) = 1.5*U*_eq_(C); C_methylene_—H = 0.97 Å, with *U*_iso_(H) = 1.2*U*_eq_(C); O—H bonds for the water molecule were constrained to 0.94 Å and 0.85 Å, with *U*_iso_(H) = 1.5*U*_eq_(O1W). Finally, the hydroxyl H atom (H2) was refined freely.

### Crystal data

**Table:**

C_11_H_10_N_4_O_4_·H_2_O	*Z* = 2
*M**_r_* = 280.25	*F*(000) = 292
Triclinic, *P*1	*D*_x_ = 1.442 Mg m^−^^3^
Hall symbol: -P 1	Mo *K*α radiation, λ = 0.71073 Å
*a* = 7.1649 (14) Å	Cell parameters from 5670 reflections
*b* = 8.2064 (16) Å	θ = 3.1–27.5°
*c* = 11.281 (2) Å	µ = 0.12 mm^−^^1^
α = 84.88 (3)°	*T* = 293 K
β = 77.70 (2)°	Prism, colourless
γ = 89.38 (3)°	0.20 × 0.18 × 0.15 mm
*V* = 645.5 (2) Å^3^	

### Data collection

**Table:**

Rigaku SCXmini diffractometer	2959 independent reflections
Radiation source: fine-focus sealed tube	1824 reflections with *I* > 2σ(*I*)
graphite	*R*_int_ = 0.040
Detector resolution: 13.6612 pixels mm^-1^	θ_max_ = 27.5°, θ_min_ = 3.1°
ω scans	*h* = −9→9
Absorption correction: multi-scan (CrystalClear; Rigaku, 2005)	*k* = −10→10
*T*_min_ = 0.965, *T*_max_ = 0.977	*l* = −14→14
6749 measured reflections	

### Refinement

**Table:**

Refinement on *F*^2^	Primary atom site location: structure-invariant direct methods
Least-squares matrix: full	Secondary atom site location: difference Fourier map
*R*[*F*^2^ > 2σ(*F*^2^)] = 0.064	Hydrogen site location: inferred from neighbouring sites
*wR*(*F*^2^) = 0.181	H atoms treated by a mixture of independent and constrained refinement
*S* = 1.02	*w* = 1/[σ^2^(*F*_o_^2^) + (0.0847*P*)^2^ + 0.0594*P*] where *P* = (*F*_o_^2^ + 2*F*_c_^2^)/3
2959 reflections	(Δ/σ)_max_ < 0.001
186 parameters	Δρ_max_ = 0.27 e Å^−^^3^
3 restraints	Δρ_min_ = −0.21 e Å^−^^3^
0 constraints	

### Fractional atomic coordinates and isotropic or equivalent isotropic displacement parameters (Å^2^)

**Table:**

	*x*	*y*	*z*	*U*_iso_*/*U*_eq_	
C1	0.7354 (3)	−0.2576 (3)	0.1023 (2)	0.0489 (6)	
C2	0.8477 (3)	−0.1276 (3)	0.13603 (19)	0.0445 (5)	
C3	0.7947 (3)	0.0281 (3)	0.16613 (19)	0.0453 (5)	
C4	0.6141 (4)	0.1200 (3)	0.1754 (3)	0.0662 (7)	
H4A	0.5476	0.1149	0.2591	0.099*	
H4B	0.5357	0.0725	0.1281	0.099*	
H4C	0.6419	0.2321	0.1451	0.099*	
C5	0.9906 (4)	0.2546 (3)	0.2222 (2)	0.0552 (6)	
H5A	0.9527	0.3352	0.1637	0.066*	
H5B	1.1266	0.2683	0.2166	0.066*	
C6	0.8858 (3)	0.2880 (3)	0.3486 (2)	0.0481 (6)	
C7	0.8471 (3)	0.4501 (3)	0.3709 (2)	0.0490 (6)	
H7	0.8787	0.5334	0.3083	0.059*	
C8	0.7616 (3)	0.4858 (3)	0.4866 (2)	0.0525 (6)	
C9	0.7117 (4)	0.3678 (4)	0.5813 (2)	0.0673 (7)	
H9	0.6549	0.3952	0.6589	0.081*	
C10	0.7481 (5)	0.2061 (4)	0.5582 (2)	0.0755 (8)	
H10	0.7139	0.1233	0.6209	0.091*	
C11	0.8352 (4)	0.1670 (3)	0.4426 (2)	0.0634 (7)	
H11	0.8597	0.0582	0.4282	0.076*	
N1	1.0337 (3)	−0.1508 (2)	0.14233 (18)	0.0543 (5)	
N2	1.0999 (3)	−0.0190 (2)	0.17513 (19)	0.0584 (6)	
N3	0.9557 (3)	0.0908 (2)	0.18882 (17)	0.0487 (5)	
N4	0.7247 (3)	0.6596 (3)	0.5081 (3)	0.0710 (7)	
O1	0.8110 (3)	−0.3876 (2)	0.07308 (19)	0.0758 (6)	
O2	0.5593 (3)	−0.2220 (3)	0.1079 (2)	0.0867 (7)	
H2	0.506 (8)	−0.295 (6)	0.091 (5)	0.18 (2)*	
O3	0.7575 (4)	0.7611 (3)	0.4204 (3)	0.1067 (9)	
O4	0.6635 (3)	0.6915 (3)	0.6118 (2)	0.1040 (8)	
O1W	0.2862 (2)	0.5822 (2)	0.09151 (18)	0.0707 (6)	
H1A	0.2667	0.5412	0.0287	0.106*	
H1B	0.1813	0.6510	0.1111	0.106*	

### Atomic displacement parameters (Å^2^)

**Table:**

	*U*^11^	*U*^22^	*U*^33^	*U*^12^	*U*^13^	*U*^23^
C1	0.0516 (14)	0.0470 (13)	0.0509 (14)	−0.0009 (11)	−0.0142 (10)	−0.0120 (10)
C2	0.0458 (13)	0.0474 (12)	0.0410 (12)	0.0008 (10)	−0.0070 (9)	−0.0130 (9)
C3	0.0468 (13)	0.0465 (12)	0.0426 (12)	−0.0009 (10)	−0.0066 (9)	−0.0113 (9)
C4	0.0578 (16)	0.0592 (16)	0.085 (2)	0.0136 (13)	−0.0185 (13)	−0.0156 (14)
C5	0.0648 (16)	0.0449 (13)	0.0547 (14)	−0.0102 (11)	−0.0053 (11)	−0.0150 (11)
C6	0.0523 (14)	0.0444 (13)	0.0492 (13)	−0.0073 (10)	−0.0112 (10)	−0.0122 (10)
C7	0.0528 (14)	0.0455 (13)	0.0500 (13)	−0.0056 (11)	−0.0120 (10)	−0.0087 (10)
C8	0.0467 (13)	0.0567 (14)	0.0573 (15)	−0.0022 (11)	−0.0113 (11)	−0.0218 (12)
C9	0.0653 (17)	0.086 (2)	0.0511 (15)	−0.0053 (15)	−0.0088 (12)	−0.0182 (14)
C10	0.093 (2)	0.077 (2)	0.0520 (17)	−0.0084 (17)	−0.0088 (14)	0.0071 (14)
C11	0.0809 (19)	0.0481 (14)	0.0610 (17)	−0.0022 (13)	−0.0148 (13)	−0.0049 (12)
N1	0.0478 (12)	0.0561 (12)	0.0629 (13)	0.0029 (9)	−0.0125 (9)	−0.0257 (10)
N2	0.0509 (12)	0.0593 (13)	0.0689 (14)	0.0023 (10)	−0.0136 (10)	−0.0255 (11)
N3	0.0495 (11)	0.0448 (11)	0.0521 (11)	−0.0024 (9)	−0.0068 (8)	−0.0163 (9)
N4	0.0608 (14)	0.0758 (17)	0.0824 (18)	0.0047 (12)	−0.0141 (12)	−0.0429 (15)
O1	0.0991 (15)	0.0526 (11)	0.0874 (14)	0.0051 (10)	−0.0357 (11)	−0.0311 (10)
O2	0.0653 (14)	0.0731 (14)	0.132 (2)	−0.0111 (11)	−0.0395 (13)	−0.0188 (13)
O3	0.144 (2)	0.0529 (13)	0.115 (2)	−0.0030 (14)	−0.0029 (17)	−0.0215 (13)
O4	0.1111 (19)	0.1131 (19)	0.0943 (17)	0.0254 (15)	−0.0156 (14)	−0.0625 (15)
O1W	0.0632 (12)	0.0561 (11)	0.0935 (14)	−0.0015 (9)	−0.0099 (10)	−0.0259 (10)

### Geometric parameters (Å, °)

**Table:**

C1—O1	1.230 (3)	C7—C8	1.374 (3)
C1—O2	1.282 (3)	C7—H7	0.9300
C1—C2	1.467 (3)	C8—C9	1.366 (4)
C2—N1	1.360 (3)	C8—N4	1.479 (3)
C2—C3	1.380 (3)	C9—C10	1.386 (4)
C3—N3	1.350 (3)	C9—H9	0.9300
C3—C4	1.479 (3)	C10—C11	1.385 (4)
C4—H4A	0.9600	C10—H10	0.9300
C4—H4B	0.9600	C11—H11	0.9300
C4—H4C	0.9600	N1—N2	1.300 (3)
C5—N3	1.466 (3)	N2—N3	1.356 (3)
C5—C6	1.510 (3)	N4—O4	1.210 (3)
C5—H5A	0.9700	N4—O3	1.219 (3)
C5—H5B	0.9700	O2—H2	0.77 (5)
C6—C11	1.377 (3)	O1W—H1B	0.9349
C6—C7	1.391 (3)	O1W—H1A	0.8483
			
O1—C1—O2	125.6 (2)	C8—C7—H7	120.4
O1—C1—C2	120.3 (2)	C6—C7—H7	120.4
O2—C1—C2	114.1 (2)	C9—C8—C7	122.6 (2)
N1—C2—C3	109.06 (19)	C9—C8—N4	119.4 (2)
N1—C2—C1	121.1 (2)	C7—C8—N4	118.0 (2)
C3—C2—C1	129.8 (2)	C8—C9—C10	118.1 (2)
N3—C3—C2	103.26 (19)	C8—C9—H9	121.0
N3—C3—C4	123.4 (2)	C10—C9—H9	121.0
C2—C3—C4	133.4 (2)	C11—C10—C9	120.4 (2)
C3—C4—H4A	109.5	C11—C10—H10	119.8
C3—C4—H4B	109.5	C9—C10—H10	119.8
H4A—C4—H4B	109.5	C6—C11—C10	120.6 (2)
C3—C4—H4C	109.5	C6—C11—H11	119.7
H4A—C4—H4C	109.5	C10—C11—H11	119.7
H4B—C4—H4C	109.5	N2—N1—C2	109.17 (19)
N3—C5—C6	114.05 (19)	N1—N2—N3	106.90 (18)
N3—C5—H5A	108.7	C3—N3—N2	111.60 (18)
C6—C5—H5A	108.7	C3—N3—C5	129.1 (2)
N3—C5—H5B	108.7	N2—N3—C5	119.26 (18)
C6—C5—H5B	108.7	O4—N4—O3	124.3 (3)
H5A—C5—H5B	107.6	O4—N4—C8	117.7 (3)
C11—C6—C7	119.2 (2)	O3—N4—C8	118.0 (2)
C11—C6—C5	123.2 (2)	C1—O2—H2	110 (4)
C7—C6—C5	117.5 (2)	H1B—O1W—H1A	102.8
C8—C7—C6	119.1 (2)		

### Hydrogen-bond geometry (Å, °)

**Table:**

*D*—H···*A*	*D*—H	H···*A*	*D*···*A*	*D*—H···*A*
C4—H4A···O4^i^	0.96	2.52	3.267 (3)	135
O1W—H1A···O1^ii^	0.85	1.94	2.755 (3)	161
O1W—H1B···N1^iii^	0.93	1.95	2.846 (3)	160
C11—H11···O3^iv^	0.93	2.57	3.423 (4)	153
O2—H2···O1W^iv^	0.77 (5)	1.88 (5)	2.592 (3)	153 (6)

Symmetry codes: (i) −*x*+1, −*y*+1, −*z*+1; (ii) −*x*+1, −*y*, −*z*; (iii) *x*−1, *y*+1, *z*; (iv) *x*, *y*−1, *z*.
